# Synthesis and Fabrication of Dialdehyde Cellulose/PVA Films Incorporating Carbon Quantum Dots for Active Packaging Applications

**DOI:** 10.3390/polym17172370

**Published:** 2025-08-30

**Authors:** Tanpong Chaiwarit, Rangsan Panyathip, Sastra Yuantrakul, Kwanjit Duangsonk, Pattaraporn Panraksa, Pornchai Rachtanapun, Kittisak Jantanasakulwong, Pensak Jantrawut

**Affiliations:** 1 Department of Pharmaceutical Sciences, Faculty of Pharmacy, Chiang Mai University, Chiang Mai 50200, Thailand; tanpong.ch@cmu.ac.th (T.C.); pattaraporn.pan@cmu.ac.th (P.P.); 2 Division of Physics, Faculty of Science and Technology, Rajamangala University of Technology Thanyaburi, Pathum Thani 12110, Thailand; rangsan_p@rmutt.ac.th; 3 Department of Microbiology, Faculty of Medicine, Chiang Mai University, Chiang Mai 50200, Thailand; sastra.y@cmu.ac.th (S.Y.); kwanjit.d@cmu.ac.th (K.D.); 4 Division of Packaging Technology, Faculty of Agro-Industry, Chiang Mai University, Chiang Mai 50100, Thailand; pornchai.r@cmu.ac.th (P.R.); kittisak.jan@cmu.ac.th (K.J.); 5 Center of Excellence in Argo Bio-Circular-Green Industry (Argo BCG), Chiang Mai University, Chiang Mai 50200, Thailand

**Keywords:** active packaging, carbon dots, composite film, dialdehyde cellulose, polyvinyl alcohol

## Abstract

Active packaging supports sustainable development by extending food shelf life and reducing spoilage, contributing to global food security. In this study, cellulose dialdehyde was synthesized and blended with polyvinyl alcohol in varying ratios to produce composite films. The incorporation of dialdehyde cellulose into films tended to increase puncture strength and Young’s modulus, decrease elongation, reduce water solubility, and enhance resistance to water vapor transmission because of crosslinking. Carbon quantum dots were subsequently incorporated into composite films to enhance their antibacterial property. This represents a novel combination of a natural bio-based crosslinker and fluorescent nanomaterials in a single packaging system. Carbon quantum dots were synthesized by an electrochemical method and incorporated as functional agents. The addition of carbon quantum dots influenced the mechanical properties of the films due to interactions between polymers and carbon quantum dots. This interaction also slightly reduced the antibacterial effectiveness of the films, consisting of dialdehyde cellulose and PVA in ratios of 3:1 and 4:0. Nevertheless, the composite films maintained sufficient antimicrobial activity against common foodborne bacteria, including *Staphylococcus aureus*, *Escherichia coli*, and *Salmonella* Typhimurium. Overall, the findings demonstrate that multifunctional material made from dialdehyde cellulose, polyvinyl alcohol, and carbon quantum dots are a promising alternative to conventional plastic packaging.

## 1. Introduction

Sustainable Development Goal 2 (SDG 2) focuses on ending hunger and achieving food security [1]. Active food packaging, which incorporates functional agents to extend shelf life and preserve food quality, helps reduce food waste and post-harvest losses. By improving food preservation and minimizing spoilage, active packaging supports the goals of SDG 2 and contributes to global food security.

Recently, packaging, especially plastics, are necessary in the modern trade of goods, and guarantee the preservation of the quality of food products. It protects packaged products from external environmental factors that can affect the quality and health safety of food products, making transportation, storage, and dispensing of products easier. Active packaging has been extensively developed to preserve and protect products by delaying oxidation, controlling respiration rate, and reducing moisture migration and microbial growth. Unlike traditional packaging, active packaging interacts with the product or its environment to extend shelf life, maintain quality, and improve safety. To address the limitations of conventional packaging, active packaging incorporates functional components such as moisture control agents, oxygen scavengers, and antimicrobials for specific intended purposes [2,3,4]. Classifications of active packaging are shown in Table 1. Antimicrobial packaging can be developed by directly incorporating antimicrobial agents into the film matrix, coating packaging films with antimicrobial substances, or producing packaging materials from antimicrobial polymers. Antimicrobial packaging films offer advantages over the direct addition of preservatives by enabling controlled and localized release of active agents onto food surfaces. This approach minimizes undesirable interactions with food components and helps preserve antimicrobial efficacy [3].

Both natural and synthetic biopolymers have been widely used as materials for active packaging in film form. Natural biopolymers such as gelatin, chitosan, starch, and cellulose are favored for their biodegradability, biocompatibility, abundance, and sustainability [5]. Additionally, some natural biopolymers exhibit antibacterial properties. For example, chitosan is an excellent biomass antibacterial material, which is widely used in the field of antibacterial materials [6].

**Figure 1:**
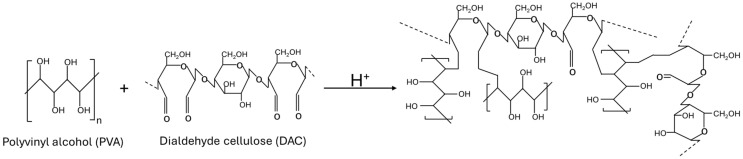
Reaction scheme of crosslinking between DAC and PVA.

Polyvinyl alcohol (PVA) is a colorless, odorless, water-soluble synthetic biopolymer with the molecular formula [C_2_H_4_O]_n_ obtained through the hydrolysis of polyvinyl acetate [7]. It is a suitable choice for active packaging due to transparency, water solubility, chemical resistance, firm forming ability, biodegradability, biocompatibility, and non-toxicity [8]. Polyvinyl alcohol (PVA) can be crosslinked using various techniques owing to its hydrophilic nature. Among the most widely applied methods are chemical crosslinking—through the incorporation of acids or bases—as well as thermal approaches. These reactions occur between the hydroxyl groups of PVA backbone and the functional groups of the crosslinking agents, leading to reduced water solubility, enhanced rigidity, and improved chemical stability of the polymer [9]. 2,3-Dialdehyde cellulose, a modified form of cellulose, can be synthesized through oxidation with periodate ions (IO_4_^−^), which selectively cleave the C2–C3 bond in the glucopyranoside ring of cellulose, converting the corresponding hydroxyl groups into two aldehyde functionalities per glucose unit [10,11]. Moreover, DAC exhibits several advantageous properties for use in active packaging materials, including low toxicity, biodegradability, biocompatibility, and antimicrobial activity against *Staphylococcus aureus* (*S. aureus*) and *Escherichia coli* (*E. coli*) [12]. The reactive aldehyde groups in DAC can react with hydroxyl groups of PVA (Figure 1) [13]. As a bio-based crosslinking agent, DAC provides biocompatibility and lower toxicity compared to conventional organic crosslinkers such as glutaraldehyde and formaldehyde [14,15]. Therefore, fabricating composite films from DAC and PVA offers a promising sustainable alternative to conventional plastic packaging.

Carbon quantum dots (CQDs), also referred to as carbon dots (CDs), are recognized as exceptional fluorescent nanomaterials due to their strong photoluminescence, excellent biocompatibility, and remarkable stability [16,17,18]. In addition, CQDs possess advantageous characteristics, including adsorption interactions, controllable surface functional groups, and nanoscale size, which enable their effective attachment to biological surfaces for antimicrobial applications [19,20]. Owing to their antimicrobial activity, antioxidant potential, low toxicity, and compatibility with a wide range of biopolymers, CQDs represent promising active agents for the development of advanced food packaging films [21,22].

Although DAC shows potential for use in food packaging, there are only a few studies exploring DAC combination with other polymers to produce composite films incorporating active agents such as CQDs. This study presents a novel approach to developing active packaging films by combining dialdehyde cellulose (DAC) as a natural bio-based crosslinker with polyvinyl alcohol (PVA) and electrochemically synthesized carbon quantum dots (CQDs). This study integrates the properties of DAC, PVA, and CQDs into a single composite system. Our study presents DAC/PVA composite films incorporating CQDs, providing a green, multifunctional, and application-oriented material for active food packaging. Consequently, this study aimed to synthesize dialdehyde cellulose (DAC), fabricate composite films with varying ratios of DAC and polyvinyl alcohol (PVA), and incorporate carbon quantum dots (CQDs) into the films for application in active food packaging.

**Table 1:** Classification of active packaging films based on function [23,24,25].

Functions	Description	Example of Active Compounds
Oxygen Scavengers	Remove residual O_2_ to prevent oxidation	Ascorbic acid, sodium sulphite, sodium metabisulfite, nanoparticles, activated carbon
Ethylene Scavengers	Absorb ethylene to slow ripening	Potassium permanganate, activated carbon, calcium oxide and ruthenium-based compounds
Moisture Control Systems	Regulate humidity to protect product quality	Bentonite clay, activated carbon, starch-based desiccants, various salts (e.g., magnesium sulphate, calcium sulphate and potassium carbonate)
Antimicrobial Packaging	Control microbial growth	Silver nanoparticles, essential oils, quaternary ammonium compounds, copper ions or compounds, antibacterial agents
Antioxidant	Delay oxidation via antioxidants or metal chelators	Alpha-tocopherol acetate, BHA, BHT, extracts of rosemary, lysozyme, cellulose acetate
Odor/Flavor Scavengers and Emitters	Manage aroma: absorb off odors or release flavors	Activated carbon, food flavors, plastic additives such as polyester, polyethylene, polypropylene, polyamide and polyvinyl chloride
CO_2_ Emitters	Modify CO_2_ levels to extend shelf life	Ferrous carbonate, ascorbic acid and sodium bicarbonate
CO_2_ Scavengers	Preventing products from becoming oxidized	Calcium hydroxide, sodium hydroxide, silica gel, potassium hydroxide and calcium oxide

## 2. Materials and Methods

Cellulose powdered 97% (REF grade) was purchased from Himedia (Nashik, India). Sodium metaperiodate (NIO_4_) was purchased from Kemaus (Cherrybrook, Australia). Polyvinyl alcohol (PVA; Mowiol^®^ 4-88, MW 31,000) was purchased from Sigma- Aldrich (Taufkirchen, Germany). Hydroxylamine hydrochloride 99% (AR grade) was purchased from Loba Chemie (Mumbai, India). Sodium hydroxide (NaOH) 1.000 M was purchased from Ajax Finechem (Seven Hills, Australia). Hydrochloric acid (HCl) 1.0 N was purchased from RCI labscan (Bangkok, Thailand).

### 2.1. Carbon Quantum Dots Preparation

CQDs were prepared by the electrochemical method, as described in previous studies [26,27,28], applying bare graphite rods as carbon precursors (99.99% purity). The graphite rods were annealed at 450 °C for 15 min to induce structural defects and remove binder components. These defects on the rod surfaces facilitated the subsequent etching and oxidation processes during CQD formation. The electrolytic solution (50 mL) was 0.1 M citric acid in deionizing water (DI water). Electrochemical exfoliation was carried out at 4 V for 5 h. The obtained CQD products were filtered through a 0.45 μm membrane and washed thoroughly with DI water.

### 2.2. Synthesis of Dialdehyde Cellulose

The synthesis of dialdehyde cellulose (DAC) was adapted from previous studies [29,30]. Alpha-cellulose (1 g) was mixed with sodium periodate (NaIO_4_, 1.65 g) dissolved in 50 mL of ultrapure water (molar ratio of anhydroglucose unit to NaIO_4_ of 1:1.25). The mixture was stirred at 30 °C and 400 rpm in the absence of light for 72 h. To terminate the reaction, 150 mL of water was added, followed by repeated washing with ultrapure water using centrifugation (MPW-352R, Warsaw, Poland) at 8000 rpm for 15 min until the solution reached neutral pH, ensuring removal of residual NaIO_4_. The collected wet DAC was subsequently freeze-dried (Christ Beta 2-8 LD-plus, Osterode am Harz, Germany) at −80 °C for 72 h. The resulting dry DAC was stored at room temperature until further use.

### 2.3. Determination of Aldehyde Content

The aldehyde content of DAC was determined following the method described by [31]. DAC powder (1 g) was dispersed homogeneously in 50 mL of deionized water, and 10 mL of hydroxylamine hydrochloride solution (5% *w*/*v*, pH 5) was added. The mixture was stirred with a magnetic stirrer at 40 °C for 4 h. The resulting solution was titrated with 0.1 M NaOH until the pH reached 5 (end point), and the volume of NaOH consumed was recorded. Alpha-cellulose was used as a control. The aldehyde content was calculated using Equation (1).

(1) $$\mathrm{Aldehyde}\;\mathrm{content}\;(\mathrm{mmol}/g)=\frac{{(V}_{1}\;-\;V_{2})\;\times \;M_{NaOH}\;}{m}$$

where $V_{1}$ is volume of 0.1 M NaOH used to tritrate DAC at the end point (mL),

$V_{2}$ is volume of 0.1 M NaOH used to tritrate alpha cellulose at the end point (mL),

$M_{NaOH}$ is concentration of NaOH (M),

m is mass of DAC powder (g).

### 2.4. Dialdehyde Cellulose Film Preparation

The preparation of composite films was adapted from [30]. Composite films were fabricated with varying weight ratios of DAC to PVA (0:4, 1:3, 2:2, 3:1, and 4:0 g, Table 2). DAC was dissolved in 94.4 g of deionized water at 80 °C for 4 h using a hotplate magnetic stirrer at 400 rpm. PVA was then added to the DAC solution and completely dissolved at 80 °C, after which the temperature was reduced to 40 °C. At this stage, 1.0 M HCl was introduced at a ratio of 150 μL of 1.0 M HCl per 1 g of PVA to facilitate crosslinking, and the reaction was allowed to proceed for 2 h. Subsequently, 1.0 M NaOH was added to neutralize the excess HCl. Glycerol (1.6 g) was incorporated as a plasticizer and stirred for an additional 30 min. For film casting, 10 g of the prepared solution was poured into a glass Petri dish (50 × 17 mm^2^) and dried in a hot-air oven at 40 °C.

For the incorporation of carbon quantum dots (CQDs), a CQD solution was directly added to the DAC/PVA mixture at a concentration of 20% *w*/*w*, ensuring homogeneous dispersion prior to the drying process.

### 2.5. FTIR Analysis

The FTIR analysis was conducted using a Fourier-transform infrared spectrophotometer (FT/IR-4700, Jasco Corp., Tokyo, Japan) with a resolution of 4 cm^−1^ in transmittance mode. Spectral measurements were recorded over the range of 500–4000 cm^−1^.

### 2.6. Absorption and Photoluminescence Analysis

The absorption properties of CQDs were observed using ultraviolet–visible spectroscopy (UV-vis, Jasco V-730 spectrophotometer) in the range of 300 to 700 nm. The fluorescence properties of CQDs were indicated by excitation at 360 nm using a spectrofluorometer (PL, Jasco FP-8650, Jasco Corp., Tokyo, Japan) to calculate the fluorescence quantum yield (QY%).

### 2.7. Morphological Characteristics of DAC Composited Films

The appearance of the films was evaluated by visual inspection. Film thickness was measured using a micrometer (3203-25A, Insize Co., Ltd., Suzhou, China). For morphological analysis, all film samples were cut, mounted onto aluminum stubs with adhesive carbon tape, and sputter-coated with gold for 1 min. The surface and cross-sectional morphologies of the films were then examined using scanning electron microscopy (SEM, JEOL JCM-7000 NeoScope^TM^, Tokyo, Japan) at 300× magnification, under low-vacuum mode, with an accelerating voltage of 15 kV.

### 2.8. Mechanical Properties Test

The mechanical properties of the films were determined using a texture analyzer (TX.TA Plus, Stable Micro Systems, Surrey, UK) equipped with a 5 kg load cell (sensitivity: 0.001 N) in compression mode. A cylindrical stainless-steel probe with a flat surface (2 mm diameter) was employed for puncture testing. Film samples were mounted on a heavy-duty platform, and measurements were performed at a constant speed of 2.00 mm/s. All tests were conducted at room temperature, with six replicates for each sample. Mechanical parameters, including puncture strength and Young’s modulus, were calculated using the corresponding equations as described by [32].

### 2.9. Film Solubility Test

The solubility of the films was determined following the method described in [33]. Film samples were cut into 10 × 10 mm^2^ pieces and dried at 105 °C until a constant weight was achieved. Their initial weight (Wi) was recorded using an analytical balance. Each sample was then immersed in 25 mL of distilled water at 25 ± 1 °C for 1, 12, and 24 h. After incubation, the films were removed, dried in a hot-air oven at 105 °C, and weighed to obtain the final weight (Wf). All measurements were performed in triplicate. The solubility value was calculated using Equation (2).

Solubility (%) = (*W_i_* − *W_f_*)/*W_i_* × 100 (2)

where *W_i_* is initial weight of sample (g),

*W_f_* is dried weight of sample (g).

### 2.10. The Water Vapor Transmission Rate (WVTR)

The water vapor transmission rate (WVTR) of the composite films was determined gravimetrically using the desiccant method [34]. The desiccant was prepared by drying calcium chloride (CaCl_2_) at 100 °C for 24 h. Dried CaCl_2_ (1.5 g) was weighed using an analytical balance and placed in plastic containers (40 mm in diameter). The composite films were placed on top of the containers and tightly sealed with Parafilm^®^. A container with CaCl_2_ but without a film cover was used as a control. All containers were kept at room temperature for 24, 48, and 72 h. At each time point, the weight of the desiccant was recorded. All tests were performed in triplicate. WVTR was calculated using Equation (3).

(3) $$\mathrm{WVTR}\;(\mathrm{mg}/{\mathrm{cm}}^{2}/h)\;=\;\frac{∆m}{A\;\times \;t}$$

where ∆m is the weight gained at each fixed time (mg),

A is an effective surface area (cm^2^),

t is the fixed time interval (h).

### 2.11. Antibacterial Activity

The agar disk diffusion method was used to evaluate the antibacterial activity of DAC composite films. All film samples were sterilized with ethylene oxide prior to testing. The antibacterial assay was adapted from [35]. *S. aureus* (ATCC 25923), *E. coli* (ATCC 25922), and *Salmonella enterica* subsp. *enterica serovar* Typhimurium (*S.* Typhimurium; IR 715) were initially cultured on tryptic soy agar (HiMedia, Mumbai, India) at 37 °C for 24 h. Subsequently, bacterial cultures were transferred to tryptic soy broth (Sigma-Aldrich, St. Louis, MO, USA) and incubated at 37 °C under aerobic conditions for 12 h. For the antibacterial test, 100 µL of bacterial suspension (OD_600_ = 0.1) was spread onto dried tryptic soy agar plates. DAC composite films (0.5 mm in diameter) were placed on the agar surface and incubated at 37 °C under aerobic conditions for 24 h. Antibacterial activity was assessed by measuring the diameter of the inhibition zone with a ruler. Clindamycin disk was used as the positive control for *S. aureus* and the positive control of *E. coli* and *S.* Typhimurium was penicillin disk.

### 2.12. Statistical Analysis

Statistical analysis was performed using SPSS software (version 17; IBM Corporation, Armonk, NY, USA) to determine significant differences between the results. A significance level of 0.05 was applied for all comparisons.

## 3. Results and Discussion

### 3.1. Carbon Quantum Dots Properties

**Figure 2:**
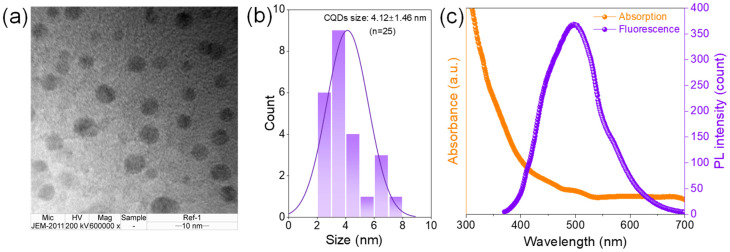
(**a**) Morphological result of CQDs, (**b**) size distribution, and (**c**) correlation of their absorption and fluorescence properties.

The obtained CQDs were characterized using transmission electron microscopy (TEM) at ×600,000 magnification, which revealed spherical-shaped nanoparticles with an average particle size of 4.12 ± 1.46 nm (Figure 2a,b). These results confirmed the achieved CQD quality. Figure 2c demonstrates the absorption edge spectrum of CQDs at 311 and 338 nm, assigning to the absorption regions of π-π* and *n*-π*, respectively [26,27,28]. The photoluminescence (PL) spectra demonstrated a maximum emission intensity at 500 nm under 360 nm excitation, yielding a quantum yield (QY) of 78.46%. These favorable optical properties confirmed the high fluorescence efficiency of the synthesized CQDs, which were subsequently incorporated into antibacterial composite films for further investigations.

### 3.2. Synthesis of Dialdehyde Cellulose and Aldehyde Content

The obtained DAC appeared as a fine off-white powder, whereas the original α-cellulose was pure white. This slight color change was attributed to the oxidation reaction with NaIO_4_. The yield of DAC was 63.98 ± 2.97% (*w*/*w*) based on the initial weight of α-cellulose. The aldehyde content of DAC was determined to be 9.88 ± 1.33 mmol/g, which was consistent with previously reported values (9.7–10.3 mmol/g) [36]. Previous studies have demonstrated that the degree of cellulose oxidation to 2,3-dialdehyde cellulose (DAC) depends on factors such as reaction temperature, oxidant concentration, and duration of reaction. Elevated temperatures generally accelerate molecular motion and enhance the frequency of molecular interactions, thereby increasing the oxidation rate [12]. However, excessive temperatures can promote the decomposition of periodate, resulting in lower aldehyde content, reduced reaction efficiency, and formation of undesirable by-products, because beyond 55 °C, periodate becomes unstable and decomposes to liberate iodine [11,37]. A high concentration of oxidant resulted in a subsequent increase in the aldehyde content but decreased the yield of DAC [11]. The reaction time significantly influenced the properties of the obtained DAC. Prolonged reaction, combined with elevated temperature and/or higher oxidant concentration in optimal conditions, tended to increase the aldehyde content. However, extended reaction time also promoted periodate decomposition and cellulose degradation [11,37]. Since periodate decomposes at elevated temperatures (>55 °C), performing the oxidation at room temperature over an extended reaction time was considered a reasonable and effective approach.

### 3.3. Determination of Aldehyde Content

The aldehyde content and yield of DAC were 9.88 ± 1.33 mmol/g and 68.43 ± 2.97%, respectively. The aldehyde content was close to the previous study reporting that aldehyde content was around 10 mmol/g [35]. In our previous study on synthesizing an aldehyde derivative from microcrystalline cellulose, the aldehyde content obtained was only 2.1 mmol/g [38]. The higher aldehyde content depends not only on reaction conditions such as reaction time, temperature, and NaIO_4_ concentration but also on the type and source of cellulose and its degree of crystallinity [11,39].

### 3.4. FTIR Analysis

**Figure 3:**
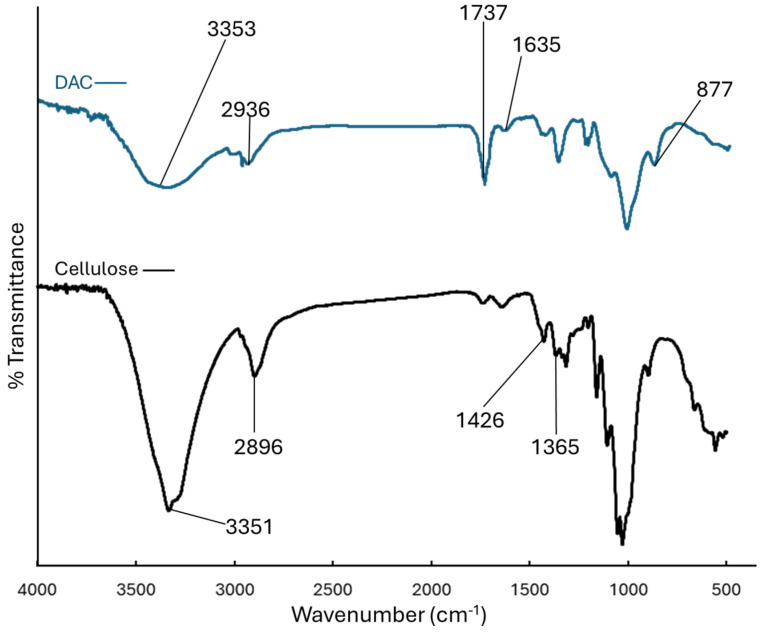
FTIR spectra of dialdehyde cellulose (DAC) and cellulose.

The FTIR spectra of DAC and cellulose are presented in Figure 3. Characteristic bands of cellulose were observed. The broad band around 3350 cm^−1^ corresponds to O–H stretching, while the peak at 2900 cm^−1^ is attributed to C–H stretching of –CH_2_ groups [29]. The peaks at 1426 and 1365 cm^−1^ represent H–C–H and O–C–H bending, as well as CH_2_ bending vibrations, respectively [29,40]. The conversion of cellulose to DAC was confirmed by the appearance of a carbonyl (C=O) stretching vibration at 1737 cm^−1^, indicating the oxidation of hydroxyl groups (–OH) in cellulose to aldehyde groups. Additionally, the peak at 877 cm^−1^ corresponds to the vibration of aldehyde groups in the semiacetal form [29,41]. The O–H stretching band at 3353 cm^−1^ became broader after periodate oxidation, further supporting the modification. A peak observed at 1635 cm^−1^ is likely attributed to aldehyde hydration, but it may also be overlapped with O–H bending vibrations [29,42].

**Figure 4:**
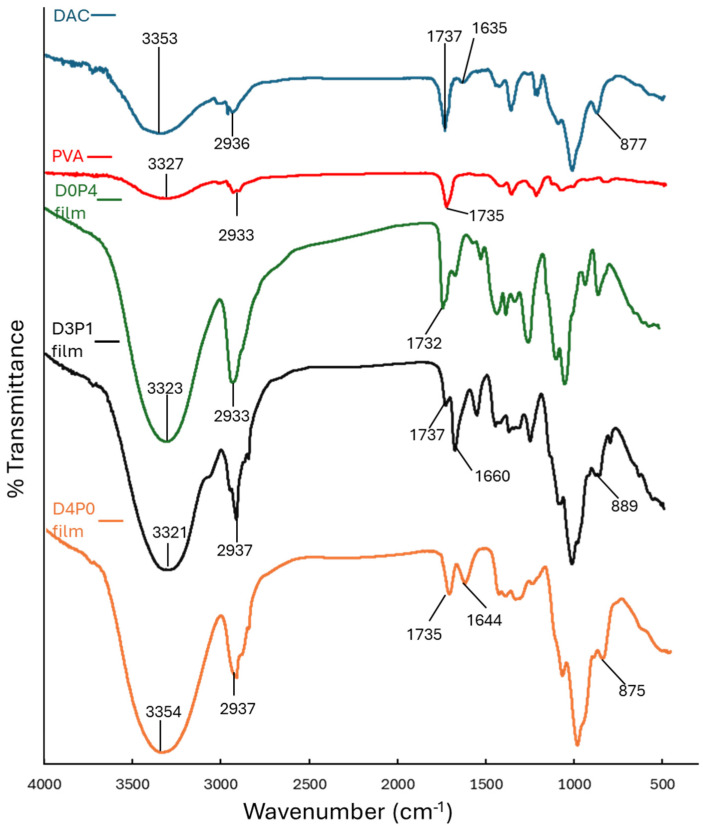
FTIR spectra of DAC, PVA, and DAC/PVA composite films.

The FTIR analysis was performed to investigate the interactions among the components in the composite films. The FTIR spectra of DAC, PVA, and the composite films are presented in Figure 4. PVA exhibited a broad O–H stretching band at 3327 cm^−1^ and a C–H stretching peak around 2933 cm^−1^. A peak at 1735 cm^−1^ was attributed to the C=O stretching of residual acetate groups remaining from the PVA preparation process [43,44]. In the spectrum of the D0P4 film, similar characteristic peaks to those of PVA were observed. The O–H stretching at 3323 cm^−1^ showed stronger intensity, likely due to water adsorption during film formation [45]. The D4P0 film also exhibits similar characteristics. The D4P0 film displayed characteristic peaks similar to DAC, with a remaining aldehyde peak at 1737 cm^−1^. A peak observed at 1664 cm^−1^ may be attributed to hydroxyl groups in cellulose adsorbing moisture, which could overlap with the aldehyde hydration peak typically expected near 1635 cm^−1^ [46]. In the D3P1 film, the O–H stretching band shifted from 3353 cm^−1^ (observed in DAC) to 3321 cm^−1^, suggesting the formation of intramolecular hydrogen bonds between DAC and PVA [44]. A peak at 1660 cm^−1^ also indicated the presence of cellulose hydroxyl groups [46]. Importantly, the intensity of the aldehyde peak at 1737 cm^−1^ decreased significantly, consistent with crosslinking between the aldehyde groups of DAC and the hydroxyl groups of PVA. The crosslinking was further confirmed by a decrease in intensity and a shift in the aldehyde hemiacetal peak from 877 cm^−1^ (DAC) to 889 cm^−1^ in the composite film [33]. By contrast, this peak appeared at 875 cm^−1^ in the D4P0 film, with no significant shift, as no interaction with PVA occurred.

### 3.5. Morphological Characteristics of DAC/PVA Composite Films

**Figure 5:**
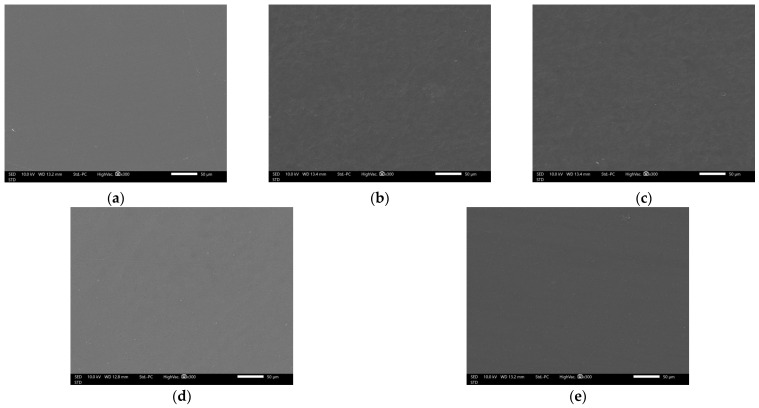
SEM micrographs showing surface of DAC/PVA composite films: (**a**) D0P4; (**b**) D1P3; (**c**) D2P2; (**d**) D3P1; (**e**) D4P0.

**Figure 6:**
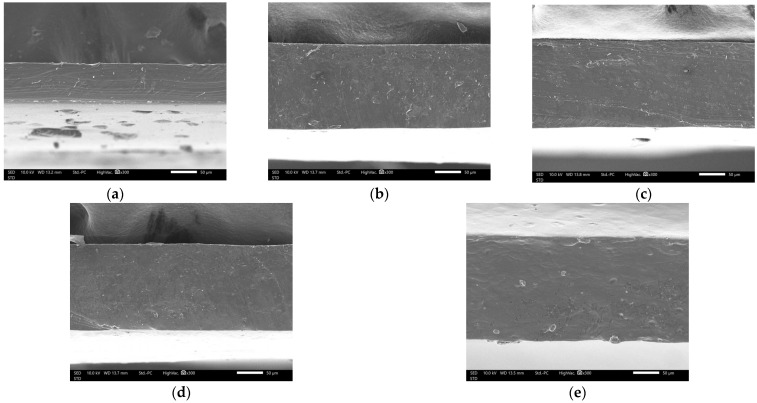
SEM micrographs showing cross-sectional microstructure of composite films: (**a**) D0P4; (**b**) D1P3; (**c**) D2P2; (**d**) D3P1; (**e**) D4P0.

The thicknesses of D0P4, D1P3, D2P2, D3P1, and D4P0, measured using a micrometer, were 0.11 ± 0.02, 0.16 ± 0.04, 0.17 ± 0.03, 0.18 ± 0.03, and 0.17 ± 0.02 mm, respectively. The thickness of the films tended to increase with a higher ratio of DAC. The SEM micrographs of the composite films are presented in Figure 5. All formulations exhibited smooth surfaces without visible particles or aggregation. Cross-sectional images (Figure 6) further revealed that all films displayed a continuous matrix without signs of phase separation between the components [33]. The SEM micrographs demonstrated that PVA and DAC were compatible and uniformly blended within the composite films.

### 3.6. Mechanical Properties Test

The mechanical properties of the DAC/PVA composite films are presented in Table 3. The puncture strength of the pure DAC film (D4P0) was higher than that of the pure PVA film (D0P4), with values of 4.52 ± 0.28 and 2.77 ± 0.43 N/mm^2^, respectively [33]. The puncture strength increased from 2.77 ± 0.43 N/mm^2^ for D0P4 to 3.89 ± 0.32 and 3.71 ± 0.27 N/mm^2^ for D1P3 and D2P2, respectively, and further increased significantly to 7.21 ± 0.63 N/mm^2^ for D3P1. Overall, the increase in DAC ratio within the composite films significantly enhanced their strength, which can be attributed to the crosslinking reactions between the aldehyde groups of DAC and the hydroxyl groups of PVA [33]. The elongation at break of the D0P4 film (86.36 ± 10.95%) was significantly higher than that of the D4P0 film (13.11 ± 4.35%), indicating that the pure PVA film was more flexible, while the pure DAC film exhibited low flexibility. As the DAC ratio in the formulations increased, the elongation at break decreased significantly from 86.36 ± 10.95% for D0P4 to 43.92 ± 6.07%, 27.07 ± 1.22%, and 5.95 ± 1.09% for D1P3, D2P2, and D3P1, respectively, due to crosslinking between PVA and DAC [33,47]. The D4P0 film showed a Young’s modulus of 22.88 ± 0.63 N/mm^2^, which was much higher than that of the D0P4 film (3.27 ± 0.52 N/mm^2^), indicating a stiffer structure. The addition of DAC to the formulations also consistently increased Young’s modulus, which rose from 3.27 ± 0.52 N/mm^2^ for D0P4 to 8.50 ± 0.65, 11.37 ± 0.82, and 54.28 ± 5.74 N/mm^2^ for D1P3, D2P2, and D3P1, respectively. The increase in Young’s modulus resulted from the formation of a stiffer polymer network caused by crosslinking between PVA and DAC [46,47]. These results suggest that PVA contributed to the flexibility of the films, while DAC enhanced their stiffness and strength.

### 3.7. Film Solubility Test

The solubility of films in water reflects their durability during application. It also determines the film’s resistance to water, oils, or other solvents. Low solubility is desirable in packaging applications intended to protect against moisture or chemical migration. The solubility values of the composite films are presented in Table 4. The D0P4 film was the most soluble, dissolving significantly within 1 h and completely within 12 h, due to the high water solubility of PVA [48]. After 1 h, the solubility of the films decreased significantly with increasing DAC ratio, from 82.44 ± 2.61% for D0P4 to 45.32 ± 2.47%, 29.86 ± 1.55%, 11.58 ± 1.24%, and 1.02 ± 0.08% for D1P3, D2P2, D3P1, and D4P0, respectively. The reduction in solubility may be attributed to crosslinking between DAC and PVA, which effectively hinders the hydrolysis of both polymers [33]. In addition, DAC is not soluble in water at room temperature but can be solubilized in hot water [36]. thereby reducing the water solubility of the composite films. Consequently, the D2P2 and D3P1 films exhibited solubility values of 20.69 ± 3.24% and 6.42 ± 1.15% after 12 h, which increased to 64.11 ± 1.70% and 10.33 ± 1.65%, respectively, after 24 h.

### 3.8. The Water Vapor Transmission Rate (WVTR)

The Water Vapor Transmission Rate (WVTR) measures the mass of water vapor permeating through a material per unit area per unit time. Effective moisture barriers are crucial in applications such as food and pharmaceutical packaging to ensure product preservation [49]. All composite films exhibited a relatively constant WVTR over 72 h. Among them, the D0P4 film showed a significantly higher WVTR at 24, 48, and 72 h (2.53 ± 0.09, 2.50 ± 0.08, and 2.44 ± 0.09 mg/cm^2^/h, respectively) compared to the composite films. This behavior can be attributed to the higher concentration of hydroxyl groups in PVA, which enhances moisture sorption due to strong hydrogen bonding with water molecules. The inherent hydrophilicity of PVA results in a greater water vapor permeation in the D0P4 film relative to DAC-containing composites [33]. The increase in DAC significantly reduced the WVTR due to a decrease in the overall hydrophilicity of the films. In particular, the WVTR values of D3P1 and D4P0 were approximately 5–5.5 and 8.4–9 times lower, respectively, than that of D0P4. Furthermore, crosslinking likely played an important role in reducing WVTR, as the hydroxyl groups of PVA interacted with the dialdehyde groups of DAC instead of adsorbing water.

### 3.9. Characteristics of DAC-Composited Films Containing CQDs

**Figure 7:**
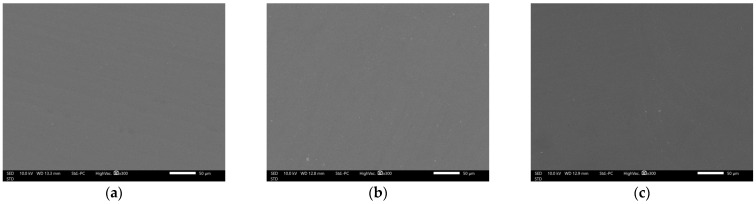
SEM micrographs showing surface of DAC/PVA composite film containing CQDs: (**a**) D0P4-CQD; (**b**) D3P1-QD; (**c**) D4P0-CQD.

**Figure 8:**
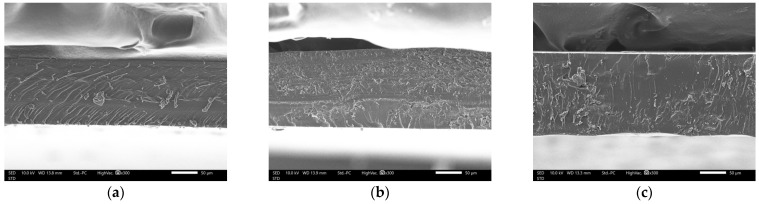
SEM micrographs showing cross-sectional microstructure of DAC/PVA composite film containing CQDs: (**a**) D0P4-CQD; (**b**) D3P1-CQD; (**c**) D4P0-CQD.

The D3P1 and D4P0 films were selected for CQDs loading and compared with the D0P4 film because both exhibited acceptable mechanical properties, lower solubility (greater integrity), and reduced WVTR (better moisture protection) compared to the other formulations. The thickness of D0P4, D3P1, and D4P0 films containing CQDs (D0P4-CQD, D3P1-CQD, and D4P0-CQD) was 0.14 ± 0.02, 0.17 ± 0.03, and 0.16 ± 0.03 mm, respectively. Compared to their corresponding films without CQDs, the thickness values of D0P4-CQD, D3P1-CQD, and D4P0-CQD were not significantly different. The SEM micrographs of the surface and cross-sectional structures of the CQD-loaded films are shown in Figure 6 and Figure 7. Similarly to the films without CQDs, D0P4-CQD, D3P1-CQD, and D4P0-CQD exhibited smooth surfaces without visible particles or aggregation, and their cross-sections displayed a continuous, homogeneous matrix (Figure 7 and Figure 8). This observation has also been reported in previous studies on polymer-based films containing CQDs, that the nanoscale size of CQDs allowed for homogeneous dispersion without significantly altering film morphology [19,50]. The addition of CQDs did not significantly alter the microstructure of the films, likely due to their nanoscale size and hydrophilic nature, which promote compatibility and uniform dispersion within hydrophilic polymer matrices [19].

**Figure 9:**
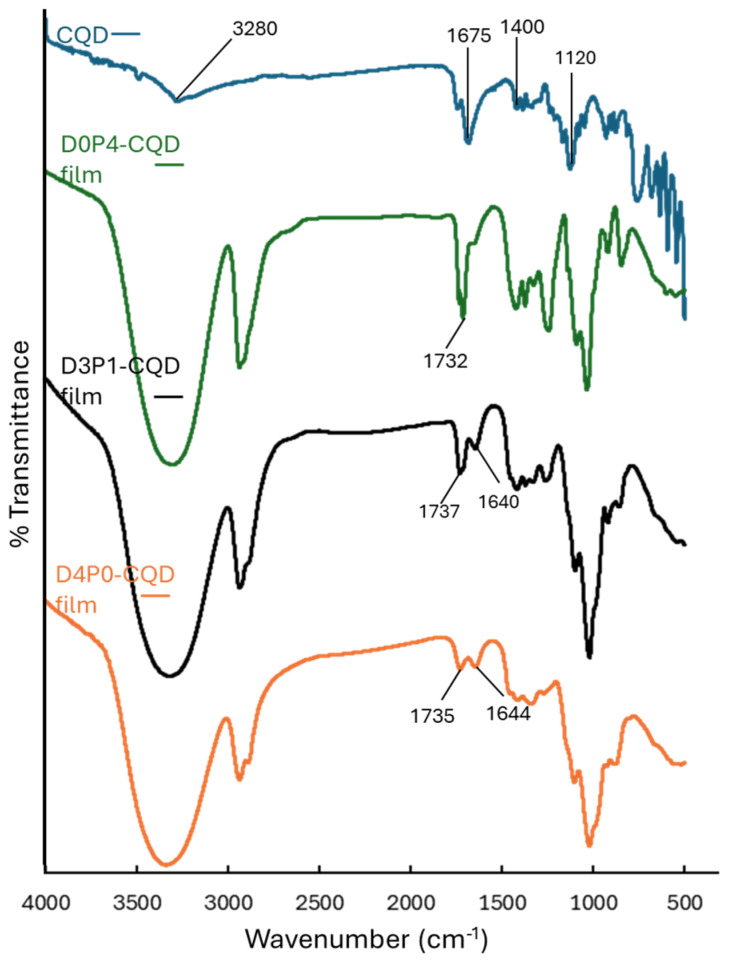
FTIR spectra of CQDs and DAC/PVA composite film containing CQD.

The FTIR spectra of CQDs and DAC/PVA composite films containing CQDs are presented in Figure 9. Characteristic absorption peaks corresponding to CQDs were observed. A broad peak at 3280 cm^−1^ was attributed to O–H stretching vibrations, indicating the presence of hydroxyl groups. The peak around 1400 cm^−1^ corresponded to symmetrical deformations of –CH_3_ groups. Additionally, the absorption band at 1675 cm^−1^ was assigned to C=O stretching of carboxylic acid groups (–COOH), while the peak at 1120 cm^−1^ was associated with C–O stretching vibrations. [51,52]. When CQDs were incorporated into the composite films, the characteristic peak of the carboxylic group (C=O stretching) at 1675 cm^−1^ disappeared. In the FTIR spectrum of the D0P4-CQD film, the characteristic carboxylic peak of CQDs was overlapped by the carbonyl peak of PVA at 1732 cm^−1^ [53]. In addition, the D3P1-CQD film exhibited decreased intensity and a shift in the aldehyde peak from 1660 to 1640 cm^−1^. Similarly, the D4P0-CQD film showed reduced intensity of the aldehyde peaks at 1735 and 1644 cm^−1^. These observations indicate interactions between the carboxylic groups of CQDs and the aldehyde groups of DAC, as well as with the hydroxyl groups of PVA and DAC.

The mechanical properties of DAC/PVA composite films containing CQDs are shown in Table 5. The addition of CQDs into the composite films affected their mechanical properties [20,54]. The puncture strength of D0P4-CQD, D3P1-CQD, and D4P0-CQD significantly decreased from the same formulation without CQDs (2.77 ± 0.43, 7.21 ± 0.63, and 4.52 ± 0.28 to 4.58 ± 0.79, 5.19 ± 0.67, and 2.41 ± 0.80 N/mm^2^, respectively). It indicated that addition of CQDs could increase the strength of D0P4 films and decrease the strength of D3P1-CQD and D4P0-CQD films. The Young’s modulus of D0P4-CQD was 8.09 ± 0.72 N/mm^2^, increasing from 3.27 ± 0.52 N/mm^2^. On the other hand, the Young’s modulus of D3P1-CQD significantly decreased from 54.28 ± 5.74 to 35.29 ± 5.80 N/mm^2^. The addition of CQDs also significantly reduced the Young’s modulus of the D4P0 film from 22.88 ± 0.63 to 18.36 ± 4.45 N/mm^2^. For PVA films, some studies have reported that incorporating CQDs increases tensile strength, owing to strong hydrogen bonding between the –COOH/–OH groups on the CQD surface and the –OH/–COOH groups in PVA chains [53,55]. Theoretically, this interaction also contributes to an increase in Young’s modulus. In contrast, for DAC and DAC composite films, CQDs could form hydrophilic interactions between their –COOH/–OH groups and the aldehyde groups of DAC, found in FTIR analysis. However, this interaction may disrupt DAC–DAC interactions, induce rearrangement of polymer chains, and interfere with crosslinking between DAC and PVA. In such cases, CQDs act plasticizer-like, resulting in a decreased puncture strength and Young’s modulus of D3P1-CQD and D4P0-CQD.

The elongation of D0P4-QD decreased dramatically from 86.36 ± 10.95% to 25.21 ± 3.12%. In contrast, the elongation at break of D3P1-QD increased significantly from 5.95 ± 1.09% to 21.27 ± 4.85%. A significant difference was also observed between the elongation at break of D4P0-CQD (13.72 ± 2.21%) and D4P0 (13.11 ± 4.35%). These results demonstrate that the addition of CQDs reduced the flexibility of D0P4 but increased the flexibility of D3P1. Previous studies have reported that the incorporation of CQDs decreases elongation of PVA films due to hydrogen bonding between the –COOH/–OH groups on the CQD surface and the –OH/–COOH groups in PVA chains [53,55]. In contrast, in the D3P1-CQD film, interactions between CQDs and DAC likely disrupted crosslinking between DAC and PVA, thereby increasing elongation at break.

### 3.10. Antibacterial Activity

The antibacterial activity of composite films is shown in Table 6. Previous studies have shown that carbon dots exhibit antibacterial activity against several bacterial strains, including *S. aureus*, *E. coli*, and *S.* Typhimurium [56,57]. The D0P4 composite film showed no antibacterial activity against *S. aureus*, *E. coli*, or *S.* Typhimurium, which can be attributed to the absence of intrinsic antibacterial properties in PVA. In contrast, the D3P1 and D4P0 composite films exhibited clear zones of inhibition against *S. aureus*, *E. coli*, and *S.* Typhimurium, which can be attributed to the antibacterial activity of dialdehyde cellulose. A previous study indicated that dialdehyde cellulose interacts with bacterial proteins and nucleic acids via Schiff base reactions, resulting in microbial inactivation [58]. This mechanism is comparable to that of glutaraldehyde; however, the dialdehyde functional groups present in cellulose possess the advantageous characteristic of nontoxicity [59]. When CQDs were incorporated, the D0P4-CQD film exhibited clear inhibition zones against *S. aureus* (7.25 ± 0.35 mm), *E. coli* (6.83 ± 0.25 mm), and *S.* Typhimurium (7.33 ± 0.29 mm). This indicated the antibacterial activity of CQD as an active compound. Bacteria consist of essential structures such as the cell wall, cell membrane, cytoplasm, proteins, and nucleoid regions. Carbon dots with antimicrobial properties can disrupt these bacterial structures; however, the susceptibility of each component to CQDs may differ [57]. Compared to D3P1, the zone of inhibition of D3P1-CQD film against *S. aureus*, *E. coli*, and *S.* Typhimurium slightly decreased from 17.00 ± 0.71 to 13.83 ± 0.29 mm, 8.00 ± 0.00 to 7.17 ± 0.29 mm, and 8.75 ± 0.35 to 7.83 ± 0.25 mm, probably because of the interaction between CQD and dialdehyde cellulose, observed in the FTIR spectra. Nonetheless, these films retained antibacterial activity suitable for applications. The D3P1-CQD film exhibited larger zones of inhibition against all tested bacteria compared to D4P0-CQD, possibly due to differences in film solubility. D3P1 film was more soluble in water than D4P0, as shown in Table 4, thus D3P1-CQD film could release more CQDs than D4P0-CQD film, when in contact with medium.

## 4. Conclusions

Carbon quantum dots, produced by an electrochemical method, exhibited a spherical nanoscale structure, maximum emission intensity at 500 nm, and high quantum yield. This study demonstrated the successful synthesis of dialdehyde cellulose and its effective blending with polyvinyl alcohol to form composite films with improved structural and barrier properties. The increase in dialdehyde cellulose proportion tended to enhance mechanical strength, reduce flexibility, lower solubility, and improve resistance to water vapor transmission because of crosslinking between aldehyde and hydroxyl groups. Carbon quantum dots were introduced into the films to impart antibacterial properties. Their addition increased mechanical strength and decreased flexibility of polyvinyl alcohol films through hydrogen bonding, while in composite films they altered the mechanical performance due to interactions with aldehyde groups. Although these interactions slightly reduced antibacterial activity, the films retained sufficient effectiveness against common foodborne pathogens such as *Staphylococcus aureus*, *Escherichia coli*, and *Salmonella* Typhimurium. Overall, this work highlights the potential of films made from dialdehyde cellulose, polyvinyl alcohol, and carbon quantum dots as sustainable active packaging materials. These films represent a promising alternative to conventional packaging, offering acceptable mechanical and barrier performance along with reliable antimicrobial protection, which contributes to extending food shelf life and improving food safety.

## Figures and Tables

**Table 2:** Formulations of DAC composite films.

Sample	Compositions			
	DAC (g)	PVA (g)	Glycerol (g)	Water (g)
D0P4	0	4	1.6	94.4
D1P3	1	3	1.6	94.4
D2P2	2	2	1.6	94.4
D3P1	3	1	1.6	94.4
D4P0	4	0	1.6	94.4

**Table 3:** Mechanical properties of DAC/PVA composite films.

Samples	Puncture Strength (N/mm^2^)	Elongation at Break (%)	Young’s Modulus (N/mm^2^)
D0P4	2.77 ± 0.43 ^a^	86.36 ± 10.95 ^a^	3.27 ± 0.52 ^a^
D1P3	3.89 ± 0.32 ^b^	43.92 ± 6.07 ^b^	8.50 ± 0.65 ^b^
D2P2	3.71± 0.27 ^b^	27.07 ± 1.22 ^c^	11.37 ± 0.82 ^c^
D3P1	7.21 ± 0.63 ^c^	5.95 ± 1.09 ^d^	54.28 ± 5.74 ^d^
D4P0	4.52± 0.28 ^a^	13.11 ± 4.35 ^e^	22.88 ± 0.63 ^e^

Different superscript letters in each column indicate significant difference (*p* < 0.05).

**Table 4:** Solubility and water vapor transmission rate (WVTR).

Samples	Solubility (%)			WVTR (mg/cm^2^/h)		
	1 h.	12 h.	24 h.	24 h.	48 h.	72 h.
D0P4	82.44 ± 2.61 ^a^	100 ± 0.00 ^a^	100 ± 0.00 ^a^	2.53 ± 0.09 ^a^	2.50 ± 0.08 ^a^	2.44 ± 0.09 ^a^
D1P3	45.32 ± 2.47 ^b^	100 ± 0.00 ^a^	100 ± 0.00 ^a^	1.81 ± 0.14 ^b^	1.76 ± 0.14 ^b^	1.72 ± 0.13 ^b^
D2P2	29.86 ± 1.55 ^c^	100 ± 0.00 ^a^	100 ± 0.00 ^a^	1.81 ± 0.08 ^b^	1.80 ± 0.12 ^b^	1.79 ± 0.11 ^b^
D3P1	11.58 ± 1.24 ^d^	20.69 ± 3.24 ^b^	64.11 ± 1.70 ^b^	0.52 ± 0.15 ^c^	0.47 ± 0.13 ^c^	0.45 ± 0.12 ^c^
D4P0	1.02 ± 0.08 ^e^	6.42 ± 1.15 ^c^	10.33 ± 1.65 ^c^	0.30 ± 0.07 ^d^	0.28 ±0.09 ^d^	0.27 ± 0.08 ^d^

Different superscript letters in each column indicate significant difference (*p* < 0.05).

**Table 5:** Mechanical properties of DAC/PVA composite films containing CQDs.

Samples	Puncture Strength (N/mm^2^)	Elongation at Break (%)	Young’s Modulus (N/mm^2^)
D0P4-CQD	4.58 ± 0.79 ^a^	25.21 ± 3.12 ^a^	8.09 ± 0.72 ^a^
D3P1-CQD	5.19 ± 0.67 ^b^	21.27 ± 4.85 ^a^	35.29 ± 5.80 ^b^
D4P0-CQD	2.41 ± 0.80 ^c^	13.72 ± 2.21 ^b^	18.36 ± 4.45 ^c^

Different superscript letters in each column indicate significant difference (*p* < 0.05).

**Table 6:** Antibacterial activity of composite films.

Sample	Zone of Inhibition (mm)		
	*S. aureus*	*E. coli*	*S.* Typhimurium
D0P4	0 ^a^	0 ^a^	0 ^a^
D3P1	17.00 ± 0.71 ^b^	8.00 ± 0.00 ^bd^	8.75 ± 0.35 ^b^
D4P0	11.00 ± 0.71 ^c^	7.75 ± 0.35 ^bde^	8.00 ± 0.00 ^c^
D0P4-CQD	7.25 ± 0.35 ^d^	6.83 ± 0.25 ^c^	7.33 ± 0.29 ^d^
D3P1-CQD	13.83 ± 0.29 ^e^	7.83 ± 0.29 ^d^	7.83 ± 0.25 ^ce^
D4P0-CQD	10.67 ± 0.29 ^c^	7.17 ± 0.29 ^ce^	7.00 ± 0.00 ^d^
Control	26.25 ± 0.43 ^d^	16.25 ± 1.52 ^f^	22.67 ± 0.52 ^f^

Different superscript letters in each column indicate significant difference (*p* < 0.05).

## Data Availability

The raw data supporting the conclusions of this article will be made available by the authors on request.

## Abbreviations

The following abbreviations are used in this manuscript:

CQDs	Carbon nano quantum dots
DAC	Dialdehyde cellulose
PVA	Polyvinyl alcohol
WVTR	Water vapor transmission rate
